# Understanding mSOS: A qualitative study examining the implementation of a text-messaging outbreak alert system in rural Kenya

**DOI:** 10.1371/journal.pone.0179408

**Published:** 2017-06-19

**Authors:** Mitsuru Toda, Ian Njeru, Dejan Zurovac, David Kareko, Shikanga O-Tipo, Matilu Mwau, Kouichi Morita

**Affiliations:** 1 Nagasaki University Institute of Tropical Medicine, Nagasaki, Japan; 2 Nagasaki University Graduate School of Biomedical Sciences, Nagasaki, Japan; 3 Japan International Cooperation Agency (JICA), Tokyo, Japan; 4 Kenya Ministry of Health Disease Surveillance and Response Unit, Nairobi, Kenya; 5 Oxford University, Oxford, United Kingdom; 6 Kenya Medical Research Institute-Wellcome Trust Research Programme, Nairobi, Kenya; 7 Kenya Medical Research Institute, Nairobi, Kenya; Dartmouth College Geisel School of Medicine, UNITED STATES

## Abstract

Outbreaks of epidemic diseases pose serious public health risks. To overcome the hurdles of sub-optimal disease surveillance reporting from the health facilities to relevant authorities, the Ministry of Health in Kenya piloted mSOS (mobile SMS-based disease outbreak alert system) in 2013–2014. In this paper, we report the results of the qualitative study, which examined factors that influence the performances of mSOS implementation. In-depth interviews were conducted with 11 disease surveillance coordinators and 32 in-charges of rural health facilities that took part in the mSOS intervention. Drawing from the framework analysis, dominant themes that emerged from the interviews are presented. All participants voiced their excitement in using mSOS. The results showed that the technology was well accepted, easy to use, and both health workers and managers unanimously recommended the scale-up of the system despite challenges encountered in the implementation processes. The most challenging components were the context in which mSOS was implemented, including the lack of strong existing structure for continuous support supervision, feedback and response action related to disease surveillance. The study revealed broader health systems issues that should be addressed prior to and during the intervention scale-up.

## Background

Disease outbreaks of emerging diseases pose serious public health risks worldwide as seen in the recent Ebola [1] and Zika [2] epidemics. Resource limited settings lack strong disease surveillance mechanisms to quickly detect, diagnose, and contain outbreaks. This hinders the nations’ abilities to fully comply with the World Health Organization’s International Health Regulations (IHR) 2005 and the Integrated Disease Surveillance and Response (IDSR) strategies [3,4]. In Kenya, as in other African countries, paper-based reports or *ad hoc* information from the health facilities reach the authorities late, which hinders abilities to take timely response actions to control the outbreaks [4]. To overcome these challenges, the Ministry of Health piloted mSOS (mobile SMS-based disease outbreak alert system) in 2013–2014. mSOS is an innovative system for health workers to report immediately notifiable diseases (required within 24 hours) in 135 health facilities in Kenya. A cluster randomized controlled trial was implemented, which showed that mSOS enhanced timely notification and that the technology can be used to enhance disease surveillance in resource-limited settings (S1 Supporting Information) [5]. Health facilities that used mSOS were able to notify immediately notifiable cases more than the facilities that did not receive the intervention (25/130, 19.2% vs. 1/39, 2.6%), and the reporting rates were significantly higher in the intervention group with percent difference of 16.7% (95% CI 2.71–25.07). Although the results were encouraging, significant gaps still remain in optimizing disease surveillance systems in Kenya. In addition, quality of implementation is rarely reported in resource-limited settings, particularly on health interventions routinely implemented by the Ministry of Health. Examining the qualities of implementation is important to better understand why certain things work and how interventions could be enhanced or modified for potential scale-up [6]. This paper reports the results of the qualitative study that examined the factors influencing the performances of mSOS implementation.

## Methods

### Study sites and participants

The qualitative study took place alongside with the mSOS trial (S1 Supporting Information) [5] at rural health facilities and sub county health management offices in the mSOS implementation areas of Busia and Kajiado counties in Kenya. Busia County borders Uganda by the Victoria Lake basin includes 7 sub counties, and Kajiado County borders Tanzania includes 5 sub counties. Participants included 11 sub county disease coordinators (SCDSC) who are the first level responders for disease surveillance activities, and 32 health facility in-charges (HFic) who are the health workers responsible for disease surveillance reporting tasks (Table 1). The selected participants were those exposed to the IDSR and mSOS intervention training, and completed in-depth interviews 6 months after the beginning of the intervention. Among 11 SCDSC, all were public health officers, 4 from Kajiado County and 7 from Busia County. Nearly all were males with the median age and posting duration of 44 and 5 years respectively. Among 32 HFic, 17 and 15 were from Kajiado and Busia counties respectively. The majority of HFic were nurses, females, and with median of 2 years working at their health facilities (Table 1).

**Table 1:** Basic characteristics of participants.

Characteristic		Sub-county Disease Surveillance Coordinator N = 11 (%)	Health facility in-charge N = 32 (%)
Kajiado County		4 (36.4)	17 (53.1)
Level of care	Hospital	NA	6 (18.8)
	Health Centre		7 (21.9)
	Dispensary/clinic*		19 (59.4)
Ownership	Public		28 (87.5)
	Private		1 (3.1)
	NGO/FBO		3 (9.4)
Gender	Female	1 (9.1)	22 (68.8)
Age, median [IQR]		44 [38–46]	39 [32–48]
Qualification	MD/CO	0	3 (9.4)
	Nurse	0	27 (84.4)
	Public Health Officer	11 (100)	2 (6.3)**
Years working at the work station, median [IQR]		5 [3–5]	2 [1–2]

CO: Clinical officer, FBO: Faith-based organization, IQR: Interquartile range, MD: Medical doctors, NA: Not Available, NGO: Non-governmental organization

*Clinic includes: one medical clinic and one maternity home

**Public Health Officer includes: one health records officer

### Intervention

The intervention was a disease outbreak alert system (mSOS), which was developed by the Ministry of Health (MOH) in collaboration with the Faculty of Information and Technology at Strathmore University in Nairobi. It was pretested and refined at several health facilities before training and deployment began in the study areas.

**Fig 1:**
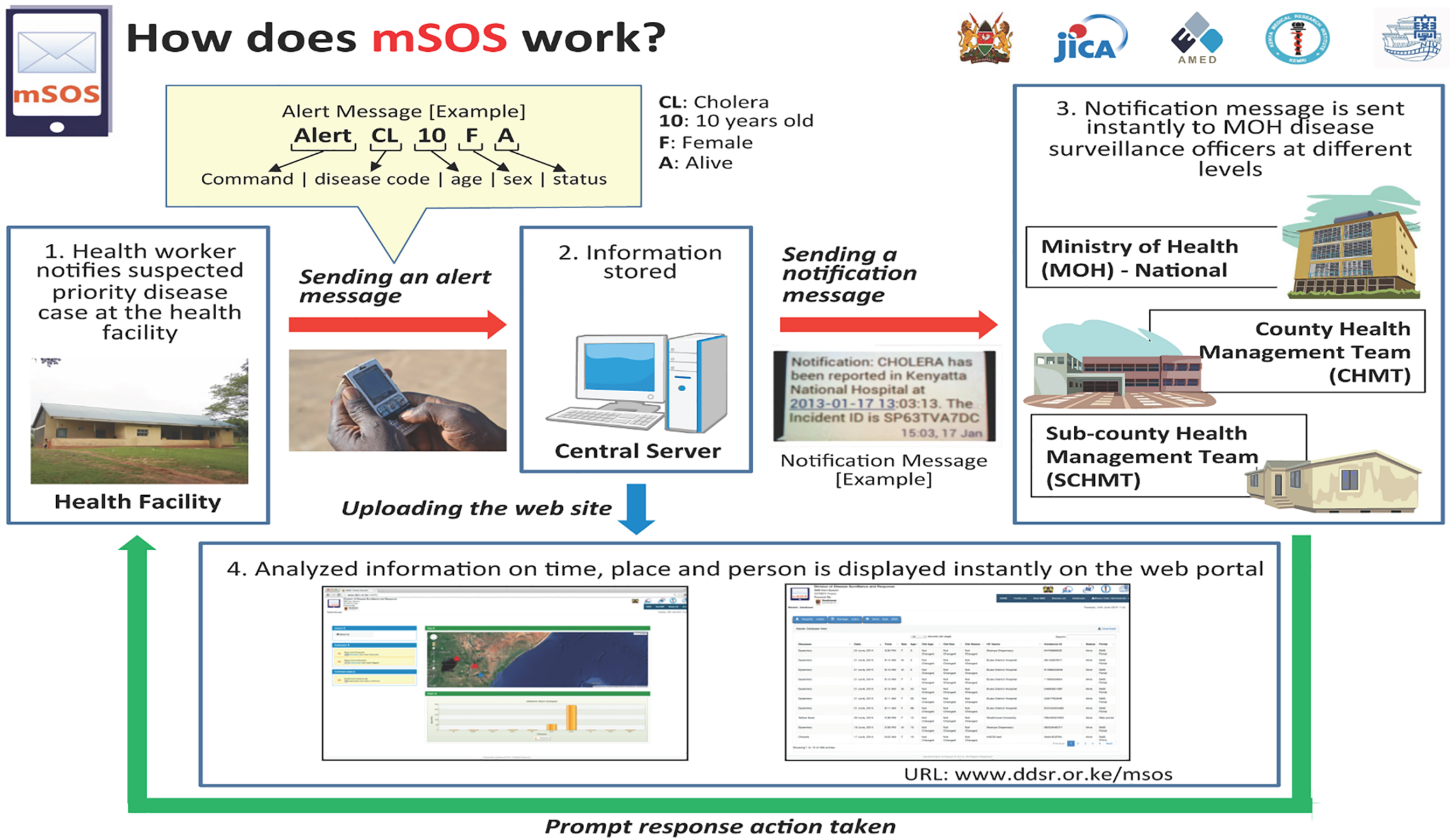
mSOS system [5].

mSOS consisted of formatted short-message-service (SMS or text messaging) communication between health workers at local facilities and MOH health managers comprising of disease surveillance coordinators at the national, county, and sub county levels (Fig 1 S1 Supporting Information [5]). Text messages sent by HFic consisted of prescribed codes specifying patients’ disease diagnosis, age, sex, and survival status (alive or dead). A web portal was developed and used to monitor notifications sent by health facility workers and response actions taken by the disease surveillance officers. By using a password protected web based portal, disease surveillance coordinators could also observe all the notifications, maps with locations of health facilities where incidents occurred, and graphs showing cumulative cases reported. HFic used a toll-free number set up by one of the telecommunication providers in Kenya. All information sent by mSOS was stored on a secure server at the MOH.

HFic used mSOS for 6 months to send patient-level information for suspected cases that required immediate notification. Twelve diseases and conditions listed in the national IDSR technical guidelines for immediately notifiable diseases (within 24 hours) were selected for the study, including Adverse Events Following Immunization (AEFI), anthrax, cholera, dengue fever, guinea worm, measles, neonatal tetanus, plague, Rift Valley fever, viral hemorrhagic fever, yellow fever, and any public health event of concern (e.g., infectious, zoonotic, foodborne, chemical, radio nuclear, or caused by unknown condition) [7].

Before mSOS was implemented in the study areas, a 1-day IDSR refresher training for all in-charges of health facilities was conducted in September and October 2013. The training focused on case definitions of notifiable disease and routine paper-based case definitions. The intervention group received an additional day of training on using mSOS for immediately notifiable disease reporting. During the mSOS training, all SCDSC were also trained on how to access and use the web based portal to view mSOS information and how to log the response actions taken. They were also guided on how to provide technical support to HFic. The Disease Surveillance and Response Unit (DSRU) at the MOH implemented all training activities.

Mobile phones were provided to health facilities and their respective SCDSC, but no airtime (telecommunication charges) compensation was given. Throughout the study period, the paper-based reporting as indicated in the national IDSR technical guidelines, continued in all study facilities. On-the-job training (OJT) of the use of mSOS by other staff than HFic was expected after the training implementation. In addition, continuous support supervision and technical assistances by SCDSC were expected in the field as per the IDSR technical guidelines. Following the mSOS and IDSR training, support supervision, technical assistance, and OJT were expected to take place at each study health facility through the routine supervisory channels.

### Data collection and analysis

Trained data collectors, supervised by DSRU, conducted interviews with participants in May 2014. The data were collected alongside with the follow-up survey for the mSOS trial [5]. The characteristics of the SCDSC and HFic managing the facility, and their exposure to the intervention were recorded.

In-depth interviews, with pre-tested interview guides, were collected on an audio recording devise and research assistants later transcribed them. The transcribed documents were imported into Nvivo 11, and analysis was conducted using the framework method [8]. The framework method allows inductive and deductive systematic analysis of qualitative data. An investigator in the study coded the transcript line by line with a research assistant according to the interview guide used in this study. Investigators in the study summarized the data into thematic areas and developed a working analytical framework. Two investigators interpreted the data based on dominant themes that emerged through the analytical framework agreed by all investigators in the study.

### Ethical approval

Ethical approval was obtained from Kenya Medical Research Institute (KEMRI) Ethical Review Committee (SSC 2523). Written informed consent was obtained from all participants.

## Results

Six dominant themes emerged from the interviews with HFic and SCDSC. The themes included: 1) experiences of IDSR and mSOS implementation training; 2) ease of use of mSOS; 3) motivation, workload, and attrition of health workers; 4) post-training follow-up, support supervision, and response action by health managers; 5) mSOS intervention and its relations to routine disease surveillance practices; and 6) views on the scale-up of mSOS.

### Experience of IDSR and mSOS implementation training

Generally, there was a vast appreciation and satisfaction of the mSOS and IDSR implementation training. Participants (22/32 of HFic and 8/11 of SCDSC) thought the training was informative and well organized. It was an opportunity for participants to learn about mSOS and refresh their knowledge on IDSR technical guidelines.

Very good because it was an eye opener very informative. HFic4, Busia County.

The training was very informative, and it was also actually exciting [because] it was the first time to be trained on mSOS. The training also acted as a…how do I put it… refresher on IDSR. And most of the facilities were sensitized. The people who are the health workers who were not trained previously on IDSR, they got that chance. SCDSC10-1, Kajiado County.

However, a few HFic (6/32 HFic) thought the training duration was short and it was hard to absorb everything on mSOS within a day. Some participants (2/32 HFic, 3/11 SCDSC) thought the training was good but short. SCDSC thought they wanted more time to learn how to use the mSOS web portal.

The training was good but with short duration to absorb information on mSOS. HFic7, Busia County.

Generally would say the time was very short. There were so many things to be covered and we would have needed more time…our time was not enough. Would have increase[d] the number of days…there was a lot still to be to be done. SCDSC8-1, Kajiado County.

### Ease of use of mSOS

Respondents (30/32 HFic and 11/11 SCDSC) had positive experiences with mSOS. HFic and SCDSC expressed views that mSOS is easy and simple to use. Participants also appreciated that mSOS was portable and accessible anywhere at any time of the day, and it only took a few minutes to send the messages.

Easy and faster to use. HFic12, Kajiado County.

It was fun. SCDSC4-1, Busia County.

You could operate from anywhere because the information would just reach you wherever you are. SCDSC5-1, Kajiado County.

A few of the technological hindrances to mSOS was network connectivity, including power fluctuation problems, network downtime, and inability to charge mobile phones because of power outages (17/32 HFic and 9/11 SCDSC).

Most of the time, we have power blackouts… SCDSC10-1, Kajiado County.

System downtime…lack of power to charge the phone. HFic10, Kajiado County.

Some of the HFic (3/32 HFic) mentioned they forgot the format on how to send text messages and had difficulties inputting age of patients under 1 year old. A few of the SCDSC (3/11 SCDSC) forgot their passwords to the web portal.

The manner in which the age of patient is to be recorded when a case is found is less than a year. Written in decimal points which is difficult to understand. HFic1, Busia County.

Challenges seen from the SCDSC’s perspective (4/11 SCDSC) was the purchase of airtime, especially in accessing the web portal. mSOS did not require airtime for health workers to report, but SCDSC needed airtime bundles to access the web portal.

You have to spend money updating your mSOS. Since we were told to update when the facility…sends such outbreak you go to mSOS to update and that is you are spending your own money. SCDSC3-3, Busia County.

Mobile phones were distributed during the mSOS intervention. Two of SCDSC (2/11 SCDSC) however reported stolen or lost phones, and there were concerns over security and handling of the phones.

In case one is away. Fear for use by colleague. HFic14, Kajiado County.

Commitment and follow up by teams delegated to continue while one is away. Handing over the phone to someone responsible posed a challenge… While one is away no one is much willing to take charge. HFic16, Kajiado County.

Somebody disappeared with one phone… SCDSC11-1, Kajiado County.

### Motivation, workload, and attrition

The simplicity of mSOS helped motivate HFic and SCDSC to notify suspected disease cases quickly (26/32 HFic and 11/11 SCDSC). Health facilities that apparently do not take part in routine disease surveillance activities also enthusiastically took part in the mSOS intervention.

Easy to report to the next level didn’t require airtime to report. HFic9, Kajiado County.

I could get some reports from facilities which never reported before. SCDSC10-1, Kajiado County.

Benefits at least I was sure of the national is receiving the alerts immediately. Not like before to alert the national it used to mean…I’m calling and the officer I’m calling is maybe in a meeting. SCDSC9-1, Kajiado County.

Although mSOS was a motivator for HFic and SCDSC, it was compounded with issues of attrition, where health worker transfers were common and often without proper handover or OJT. Factors hindering or compromising the use of mSOS also included workload and inadequate human resources (6/32 HFic, 6/11 SCDSC).

Ok in facilities there are those turnovers…constant… you will find that in some areas totally there is nobody who is now able to…send…that information, that report…so you need to re-train people…so that that it is not only one person that is reporting such that in case they go away, the surveillance activity is going to continue. Ok if you look at a facility where we have only one nurse, and she was the one that was trained and she is being transferred, who else would…still be there to really continue? Most of them we have one nurse, just a nurse…At times they are closed, they leave the…. report to the …subordinate. At times, the subordinate doesn't even know how to report. So basically things go down. SCDSC8-1, Kajiado County.

[Only] one staff [is] managing facility. While [the facility in-charge is] away, cases may be missed [and] no reporting [will be done]. HFic9, Kajiado County.

Doing on [the] job training was not easy. All health workers should be trained. HFic1, Busia County.

Because of the workload, [health workers] need motivation… we need to motivate our people… SCDSC8-1, Kajiado County.

### Post-training follow-up, support supervision, and response action

Support supervision and follow-up are necessary at the health facility level.

I think we need to do a lot of follow-ups in the facilities so that even at the [sub county] level so that almost every staff… is able to use this system in such a way…the facilities can respond. SCDSC8-1, Kajiado County.

Unfortunately, HFic (10/32 HFic) mentioned the feedback and support supervision practices did not improve after the training.

Yes reporting was on time even though there was no action. HFic3, Busia County.

It shortened the notification time but did not change anything in response. HFic8, Busia County.

The notification was done immediately response action was never communicated and was never done. HFic5, Busia County.

We are becoming serious [in] what needs to be done because [mSOS] was something it was new. We need to do a lot of follow ups… People were supposed to be…kept on toes now and then we need to move to their facilities every now and then…look at their challenges, look at their achievement[s], and all that so that they get more motivated and more internalized in doing all…what…are required…I think things would have really improved. SCDSC8-1, Kajiado County.

There was a sense that managers were not taking disease surveillance seriously (SCDSC 3/11), which in turn demotivated HFic and SCDSC to continue using mSOS.

The technology is good but let the higher levels take it seriously otherwise there is no need of sending the instant reports. The last time is sent a suspected tetanus baby but he died without anyone coming to see the child at the facility I had referred to. HFic13, Kajiado County.

You will notice that there is, there is an outbreak…Ok fine getting to know that there is an outbreak…but the county is not supportive in any way… Take for example. . . . since we realized we had aflatoxicosis in this place it is now two weeks running and they have done nothing…You are there staring at a problem that nobody takes very seriously…whenever I send any alerts in the to the national, I send that also to the county, so they are aware. SCDSC8, Kajiado County.

Feedback and supportive supervision is a cornerstone of disease surveillance, but some of the SCDSC were rarely part of the routine supervisory visits at rural health facilities (3/11 SCDSC).

Maybe…at times when there is support supervision, we are not included the DDSC [SCDSC] at times is not included in this team. And which it is crucial for a DDSC [SCDSC] to be included in support supervision because, some of these problems they normally encounter during support supervision can be rectified. SCDSC7-1, Busia County.

Other reasons of lack of support supervisions included issues of transportation and funding (10/11 SCDSC), which were not adequately provided by sub county or county offices. In addition, accessibility of rural facilities was commonly voiced as a major hindrance.

The other challenge is transport… in terms of fuel. The vehicle is there and the motorcycle is there but getting fuel sometimes is a challenge, you see like nowadays there is a very complicated protocol. SCDSC10-1, Kajiado County.

You realize we are going to our own pockets to maintain those motorcycles…and maybe lunches during supervision. SCDSC4-1, Busia County.

Issue to do with transportation. I mean something like lack of fuel because you can be called for an emergency or you would like to go for supervision on your own… So you cannot write to the district because the district also they don’t have enough funds to go out [to the field]. SCDSC2, Busia County.

The impassable roads like sometimes most of …like during the floods time the terrain could not be passable. SCDSC5-1, Busia County.

### mSOS intervention and its relations to routine surveillance and IDSR guidelines

A number of issues related to disease surveillance became apparent through the implementation process of mSOS. Health facilities often lacked paper-based tools, and health workers had sub-optimal understanding of IDSR guidelines and standard case definitions (10/32 HFic, 6/11 SCDSC).

No we do not have the forms. HFic13, Kajiado County.

Yes we always fill [case based forms] but no one follows up so we got discouraged and stopped. HFic7, Busia County.

Challenge is the standard case definition you will find that a facilities is reporting on mSOS there are issues that are not meeting standard case definition. SCDSC1-1, Busia County.

Clinicians should be sensitized on the use of case definitions so that reporting can be … accurate as well. HFic12, Kajiado County.

Participants supported abandoning the paper-based tools and integrating IDSR weekly reporting tools to mSOS (9/32 HFic, 6/11 SCDSC).

Reporting tools are very many and need to be integrated. HFic17, Kajiado County.

Would want all the reporting to be done through mSOS including the [IDSR] reports. HFic15, Kajiado County.

Notification to national level was fast compared to sending hard copies… Reduces paperwork, timely in reporting and receiving response action before there is an outbreak. HFic4, Busia County.

The information is relayed to national level instantly. Can replace the hard copies (paper work)…Fast system results based, convenient, should replace paper work. HFic2, Busia County.

### Views on the scale-up of mSOS

Despite challenges encountered with the use of mSOS, almost all respondents were enthusiastic about scale-up (30/32 HFic, 11/11 SCDSC). There was a sense that mSOS would help contain eventual disease outbreaks and it would help improve the current disease surveillance practices (9/32 HFic, 9/11 SCDSC). Users thought that the system was reliable because it reduced the need for multiple officers entering and sending the data up the chain. In addition, there was a sense that notification messages were sent to the right people at the right time (6/32 HFic). Furthermore, there was a general sense that mSOS increased notification and response rates, information was shared and compared across sub counties and counties, and disease occurrences were mapped countrywide (6/11 SCDSC).

Alerts are sent immediately…thus reduce a lot of mortalities. HFic5, Busia County.

It can be used easily in hard to reach areas. HFic16, Kajiado County.

It makes surveillance a bit easier…because from each facility or whoever has the same mSOS phone… is able to report what is happening in his facility…then it comes to me directly… then it will require for my attention… once I also receive it I just know okay… so efficiency in surveillance was enhanced… SCDSC4-1, Busia County.

Fast in sending and receiving notification, message to the national level will reach intact and minimize distortion if it were to go through many hands via the district. HFic6, Busia County.

As you are aware right now is an outbreak of Ebola in these most of these Western … Western countries of Africa, West Africa… So when we receive any case of Viral Hemorrhagic Fevers…around, then we can exchange very fast, ideas very fast. SCDSC7-1, Kajiado County.

Because our core mandate as the [SCDSC] is to…ensure that the disease does not spread, from one locality to another, and also for quick action to be taken for that disease not to spread… if [mSOS] is rolled out countrywide, the [SCDSC] may be able to… receive information every fast and act very fast…so that we can…reduce… [the] number of communicable diseases in our country…on time, because if we have to wait for a week in order to get the weekly reports… by the time…one week ends, the disease has spread to so many parts of the country…SCDSC7-1, Busia County.

I recommend it. It is very convenient and it should be rolled-out…to all over the country. It will go a long way in combating these disease outbreaks. SCDSC10-1, Kajiado County.

I will support that it is [a] good idea and [if] we [can] increase the number of diseases…generally it should cover all IDSR…targeted diseases and I think it is something that is workable. I believe [mSOS] has improved our general performance and general view of IDSR as a system…detection and response of our emergency…in terms of detection and response. SCDSC8-1, Kajiado County.

## Discussion

The quality of the intervention implementation is an important determinant for the success of any project. Our examination of mSOS intervention revealed series of successes. mSOS was perceived to be a good innovation, the technology was well accepted and SCDSC and HFic generally thought that mSOS enhanced knowledge and motivation. The results showed the potential complexity of mobile technology implementation. The technological aspect was a minor implementation issue, while the major challenge was the health system context in which the innovative technology was implemented. Our study reveals a number of issues that health systems researchers, policy makers, and implementers should take into account when designing or expanding similar interventions in resource-limited settings.

In our study, as also shown in other text messaging interventions [9,10], participants largely accepted the technology, confirmed its simplicity and ease of use, and appreciated sending and receiving outbreak information on their mobile phones through a toll free number. We do however acknowledge that the distribution of mobile phones was probably an unnecessary component to increase health worker motivations. We observed lost or stolen mobile phones without having resources to replenish. Similar challenges have been observed in a study in Swaziland [11]. For potential scale-up, we recommend in a country such as Kenya where health workers universally possess personal mobile phones [12], to leverage this opportunity. With respect to motivation and sustained performance, enhancement of managerial response and supportive supervision may be more relevant than phone distribution.

Training implementation was perceived to be informative and well organized. However, challenges in IDSR implementation as previously reported on a smaller scale in Kenya [13], were observed. In our setting, general IDSR knowledge only increased from 40% to 60% as measured in pre and post training knowledge assessment (unpublished data). One of the possible reasons for unrealized full potential of the training is its insufficient duration. Similar observation has been made in other studies and longer training was suggested to be more effective in improving health worker performance [14]. Implementers also need to be aware that participants often prefer longer seminar days because of extra per diem or stipends. Modalities of future training should however take into account quality, quantity, health worker desires, and resources available for the training.

Lack of support and reinforcement by implementers through the post training follow up and support supervision were revealed as weak components of this intervention. Effectiveness of stand alone intervention, such as the one that was *de facto* implemented in our study, is likely to be compromised unless the system is fully integrated into the routine national surveillance system with adequate resources supporting close post training monitoring of performances, continuous OJT, and supportive supervision [15–17]. These challenges compounded with lack of reporting tools and general resources (eg. transportation, airtime) have been commonly observed in Kenya as well as in other studies implementing IDSR [18–27] and IHR [28,29]. Disease surveillance activities are unfortunately not seen as priorities, neither for health workers nor for managers [30]. Innovative strategies, such as institutionalizing IDSR strategies within training colleges, are needed to prioritize IDSR as a national strategy and to be fully implemented within the health sector in Kenya and other sub-Sahara African countries.

What should also be acknowledged here are the generic and complex intertwined health systems issues that were heavily present in our study. Success of mSOS was compounded by high turnover of peripheral health workers, which is a common challenge in Kenya and in other developing countries [31–33]. This was particularly enhanced during the study period taking place during the devolution process in Kenya, which resulted in the creation of new 47 administrative units and counties [34].

Despite unfavorable timing of our study and the challenges encountered, all respondents were supportive of mSOS. To our view, this is partly based on the expectations of what the technology can accomplish in the future. We recommend gradual expansion accompanied with significant focus on the quality of implementation, which would primarily address the issue of ongoing and sustained support of health workers and surveillance coordinators in rural areas.

There are several limitations to this study. First, we cannot rule out courtesy bias where health workers have mentioned more positive results to continue the intervention. This may have been augmented by the inclusion of national MOH managers included as part of the data collection teams. However, common negative tones resonating through the study diminish the possibilities of such bias. Second, the post-intervention survey took place 6 months after the training, and there may have been recall biases. Finally, data collection was conducted together with a quantitative study. This approach facilitated the interviews and to obtain a relatively large sample size, but at the same time, we could not go in-depth on certain questions without the prior knowledge on the performance at each facility.

## Conclusions

The study highlights that innovative disease surveillance using mobile technology is well accepted, and its scale-up highly promoted by health workers and managers. The study also shows that the broader health system issues, such as post training follow-up, supportive supervision, and prioritization of disease surveillance, need to be addressed prior to and during the national rollout. Further research on understanding the challenges of IDSR and IHR compliances to enhance disease surveillance in Kenya is justified.

## Supporting information

S1 Supporting Information Toda M, Njeru I, Zurovac D, Tipo SO, Kareko D, Mwau M, et al. Effectiveness of a mobile Short-Message-Service-Based Disease Outbreak Alert System in Kenya. Emerging infectious diseases. 2016;22(4):711–5. 10.3201/eid2204.151459. 26981628; PubMed Central PMCID: PMC4806970.
(PDF)
